# Comparative immunopathogenesis in a murine model of inhalative infection with the mucormycetes *Lichtheimia corymbifera* and *Rhizopus arrhizus*

**DOI:** 10.1371/journal.pone.0234063

**Published:** 2020-06-17

**Authors:** Günter Rambach, Verena Fleischer, Verena Harpf, Michaela Lackner, Andreas Meinitzer, Hans Maier, Johannes Engesser, Cornelia Lass-Flörl, Cornelia Speth

**Affiliations:** 1 Institute of Hygiene and Medical Microbiology, Medical University of Innsbruck, Innsbruck, Austria; 2 Christian Doppler Laboratory for Invasive Fungal Infections, Innsbruck, Austria; 3 Clinical Institute of Medical and Chemical Laboratory Diagnostics, Medical University of Graz, Graz, Austria; 4 INNPATH GmbH–Institute of Pathology, Innsbruck, Austria; University of Minnesota, UNITED STATES

## Abstract

Pathogenic mucormycetes induce diseases with considerable morbidity and mortality in immunocompromised patients. Virulence data comparing different Mucorales species and various underlying risk factors are limited. We therefore compared the pathogenesis of inhalative infection by *Rhizopus (R*.*) arrhizus* and *Lichtheimia (L*.*) corymbifera* in murine models for predominant risk factors for onset of infection. Mice with diabetes or treated with cyclophosphamide or cortisone acetate were challenged via the intranasal route with an isolate of *R*. *arrhizus* or *L*. *corymbifera*, *respectively*. Clinical, immunological and inflammation parameters as well as efficacy of posaconazole prophylaxis were monitored over 14 days. Whereas immunocompetent mice showed no clinical symptoms after mucormycete infection, mice treated with either cyclophosphamide (CP) or cortisone acetate (CA) were highly susceptible. Animals infected with the isolate of *R*. *arrhizus* showed prolonged survival and lower mortality, compared to those exposed to the *L*. *corymbifera* isolate. This lower virulence of *R*. *arrhizus* was risk factor-dependent, since diabetic mice died only after infection with *Rhizopus*, whereas all *Lichtheimia*-infected diabetic animals survived. Under posaconazole prophylaxis, both mucormycetes were able to establish breakthrough infections in CA- and CP-treated mice, but the course of infection was significantly delayed. Detailed analysis revealed that susceptibility of CA- and CP-treated mice could not be mimicked by exclusive lack or downmodulation of neutrophils, platelets or complement, but can be supposed to be the consequence of a broad immunosuppressive effect induced by the drugs. Both *Lichtheimia corymbifera* and *Rhizopus arrhizus* induce invasive mycoses in immunocompromised hosts after inhalative infection. Key parameters such as virulence and immunopathogenesis vary strongly according to fungal species and underlying risk group. Selected neutropenia is no sufficient risk factor for onset of inhalative mucormycosis.

## Introduction

Mucormycosis, induced by fungi of the order Mucorales, is worldwide an emerging fungal infection and therefore gains more and more attention. Studies reported an incidence in the general population of 0.5–1.2 cases per million inhabitants per year [1]. Among the most common agents of mucormycosis worldwide and in Europe are the genera *Rhizopus spp* and *Lichtheimia spp* [2].

Most cases of mucormycosis originate from inhalation of fungal spores and present with rhino-cerebral or pulmonary manifestations. Other common presentations are cutaneous mucormycosis after skin trauma or dissemination [3]. Due to the angioinvasive capacity of mucormycetes, all these manifestations can come along with extensive thrombosis and ischaemic tissue necrosis [4].

Immunosuppression is the central risk factor to acquire mucormycosis, and medical advances e.g. in organ transplantation, treatment of cancer patients or patients with severe burns also resulted in increasing numbers of patients at risk [5, 6]. Hematological malignancies are the dominant underlying risk factors in high-income countries, while uncontrolled diabetes mellitus and skin trauma predominantly cause risk in developing countries, especially India [2, 7].

Mucormycosis is characterized by a high morbidity and mortality, due to delayed diagnosis as well as to resistance against voriconazole and echinocandins [3, 8]. To improve the outcome of infected patients, a more detailed knowledge in immunopathogenesis is strongly demanded. Of particular interest is the comparison between different Mucorales species to establish more precise virulence profiles of this heterogeneous group and to obtain better data about the relevance of the underlying risk factors. Furthermore, a clear picture of the role of defined innate immune elements might open the possibility of a targeted immune-based therapy.

## Materials and methods

### Antibodies, media and chemicals

All antibodies were purchased from BioLegend (San Diego, CA, USA). SUP (Supplemented Minimal Medium) was prepared with yeast extract (Roth) supplemented with glucose, NH_4_Cl, KH_2_PO_4_, K_2_HPO_4_ (Roth) and MgSO4 (Merck) [9]. Streptozotocin was purchased from Sigma, blood glucose test strips from Acon Diabetes Care and urine test strips from Promeditech.

### Fungal isolates and cultivation

The strains of *Lichtheimia corymbifera* (CBS 109940) and *Rhizopus arrhizus* (CBS 126971) are patient isolates, derived from clinical specimen and confirmed by sequencing the internal transcribed spacer (ITS) region of the ribosomal genes [10].

The fungal isolates were grown on SUP agar for 5–7 days at 37°C until sporulation was clearly visible. Spores were freshly harvested by rinsing with PBS with 0.05% Tween-20 (Sigma-Aldrich), washed with 0.9% NaCl and filtered through a 45 μm and a 10 μm cell strainer (BD Diagnostics System). After counting with a hemocytometer, the spore suspension was diluted in 0.9% NaCl with 0.05% Tween-20 and used immediately for intranasal infection of the animals. Stock solutions were preserved at 4°C or, for longer time periods, frozen at -80°C.

### Animal experiments

#### Ethics statement

The Central Laboratory Animal Facility of Medical University of Innsbruck and all experimental procedures of the study were complied with the Austrian Animal Experimental Act (BGBl. I Nr. 114/2012). All animal experiments were approved by the National Committee for Animal Care of the Austrian Federal Ministry of Science, Research and Economy (BMWFW) (approval numbers BMWFW-66.011/0110-WF/V/3b/2016 and BMWFW-66.011/0180-WF/V/3b/2016). Blood sampling and euthanasia of mice were performed under isoflurane anesthesia, and all efforts were made to minimize suffering.

#### Housing, husbandry, humane endpoints

The experiments were conducted using 7-week-old female C57/Bl6J mice (Charles River Laboratory, Sulzfeld, Germany). Mice were housed in polyetherimide cages with laboratory bedding, nesting material, gnawing sticks (ABEDD, Austria) and mouse shelters (EHRET, Austria). In the housing room, air exchange rate was adjusted to 12times/hour; temperature was maintained at 22°C. Mice were fed with normal mouse chow (pellets, ssniff laboratory diets, Germany) and water ad libitum.

Experimental groups each included six mice, except for the diabetes groups with nine animals to guarantee sufficient numbers of animals exhibiting hyperglycemia. All experimental procedures were conducted by scientists who attended special training by veterinarians of the Central Laboratory Animal Facility of the Medical University of Innsbruck.

Defined humane endpoints included body weight loss of more than 20%, decrease in the average body surface temperature of more than 2.5°C, disturbed or missing feeding/drinking, dyspnea (inspiratory/expiratory stridor, agonal respiration), and neurological symptoms such as abnormal body posture (body/head tilt), impaired mobility, walking in circles or body rotation. The latter symptoms indicate affectation of the central nervous system. Each mouse meeting an endpoint criterion was immediately anesthetized by isoflurane inhalation and euthanized by cervical dislocation. No animals died before meeting the criteria.

#### Performance of animal experiments

To compare different regimens of immunosuppression, 300 mg/kg of cortisone acetate were administered intraperitoneally on day -4 prior to infection and subsequently every 3^rd^ day to maintain prolonged immunosuppression. Similarly, 100 mg/kg cyclophosphamide were injected i.p. according to the same time schedule. Immunocompetent mice were mock-treated with physiological saline solution until infection. To induce insulin-dependent ketoacidosis, animals were intraperitoneally treated at day -10 with 110 mg/kg streptozotocin in 200 μl citrate buffer (0.1 M, pH 4.5, sterile-filtrated) after 4 h of fasting. Development of hyperglycemia was evaluated by measuring the blood glucose concentration with glucose test strips 5d after streptozotocin treatment; furthermore, glucose levels in urine were analyzed by urine test strips. Animals that did not develop hyperglycemia (blood glucose levels <150 mmol/l) were re-injected with 70 mg/kg streptozotocin; blood and urine glucose levels were controlled again at day -1. Animals not showing hyperglycemia were excluded from the experiment before infection. Persistence of hyperglycemia was likewise determined whenever blood was taken from the animals during and at the end of the experiment.

In one experiment aiming to study antimycotic prophylaxis, posaconazole (25 mg/kg) was administered orally by gavage, starting at day -5 prior to infection and continuing once daily until end of the experiment or until the mice met defined humane endpoints. In this experiment, diabetic mice and mice treated with cyclophosphamide or cortisone acetate as described above were included.

Another experiment aimed to study the relevance of single innate immune elements. For this purpose, neutropenia was induced by intraperitoneal application of a monoclonal blocking Ly6G antibody (clone 1A8) [11] at day -2, day 1 and day 4. This antibody decreases the neutrophil number for more than 90% at least for 3 days after each injection. Thrombocytopenia was triggered by intraperitoneal application of a rabbit α-mouse platelet serum (Accurate Chemical) every other day throughout the experiment, starting at day -1; blood count and FACS analysis confirmed depletion of more than 80% of platelets. To avoid resistance against this antibody, we switched to a rat-α-mouse CD41 antibody at day 7 with same schedule and same efficiency of platelet depletion. Mice deficient in complement factor C3 were purchased from Charles River Laboratory.

At day 0, mice were either mock-treated or infected intranasally with *Lichtheimia corymbifera* or *Rhizopus arrhizus*, respectively. For the first experiment, 6×10^6^ or 1.2x10^7^ colony forming units (CFU, viable spores) were used; for the posaconazole and innate immunity experiments 2x10^7^ CFU. Body weight, body temperature (non-contact surface thermometer, Geratherm) and clinical status were monitored at least twice daily (up to two-hourly intervals for symptomatic animals) over a period of 14d. Blood was taken at day 1 and 7 after infection as well as at the day of exitus (either at day 14 after infection or whenever an animal met humane endpoints).

#### Analysis of blood and organ samples

Whole blood (100–150 μl) was taken from the submandibular vein of the animals at indicated time points after infection using EDTA as anticoagulant. Hemograms were determined by a blood cell counter (Animal blood counter Vet abc, Scil). To measure the CFU counts in blood, 10 μl of whole blood were diluted 1:10 in 0.9% NaCl, plated on SUP agar and cultured for 48 h at 37°C.

Organs (spleen, kidneys, liver, lung and brain) were dissected after sacrification of the animals and examined macroscopically. Most organs were frozen in liquid nitrogen and stored at -20°C until PCR was performed. Some organs were fixated in 4% formalin; sections of the organs were subsequently stained with a combined hematoxylin and eosin/methenamine silver stain [12].

### Detection of Mucorales in lung and brain by polymerase chain reaction

#### Mechanical lysis

For cryo-homogenization of tissues and DNA extraction the organs were placed into sterile sample bags (Whirl-Pak®, VWR, Vienna, Austria) and frozen in liquid nitrogen. After mechanical disruption into smaller pieces the tissue was transferred in a pre-cooled reaction tube (Eppendorf, Hamburg, Germany), containing one steel grinding ball (Ø 5 mm) and homogenized in three intervals at 30 hz for 30 seconds using the MixerMill MM400 (Retsch, Haan, Germany).

#### DNA extraction

DNA was extracted from aliquots of 20 mg of homogenised tissue using the DNeasy Blood & Tissue Kit (Qiagen, Hilden, Germany) according to manufacturer’s instructions. The incubation time was set at 2 hours at 56°C, 400 rpm (Thermomixer compact, Sigma-Aldrich, Vienna), including vortexing every 15 minutes to improve enzymatic lysis.

#### qPCR

The qPCR was performed with CFX96 (BioRad, Vienna, Austria) using SsoFast™ EvaGreen® Supermix (BioRad, Vienna, Austria) in a total volume of 20 μL, containing 10 μL of EvaGreen® Supermix, PCR-grade water, 2 μL template DNA and 500 nM primers (TEF_468Fw: 5‘-GGAAGTTCGARACCCCCAAG–3‘; TEF_640Rv: 5’-CGGGTTTGACCRTCCTTGGA–3’). Primers were previously designed for housekeeping gene TEF (translation elongation factor) and resulting in a small fragment sized 171 bp. Primers were *in silico* tested for cross-reactivity with human, murine, virus, bacteria and fungi using NCBI blast comparison tool (https://blast.ncbi.nlm.nih.gov/). Specificity tests were conducted with 10 ng of fungal DNA for: *Candida albicans*, *C*. *glabrata*, *C*. *parapsilosis*, *Scedosporium prolificans*, *Aspergillus terreus*, *A*. *fumigatus*. Limit of detection (LoD) for the PCR reaction was previously found to be 1 genome copy/reaction. The PCR was done with the following conditions: initial denaturation at 95°C for 5 min, followed by 45 cycles using 95°C for 15 sec, 61°C for 30 sec and 72°C for 30 sec. Melt curve analysis was set from 60°C to 95°C with an increment of 0.5°C for 5 sec. Each run included a 5-point standard curve, a positive control, a negative control, and a NTC (no template control) performed in triplicates; samples were analyzed in duplicates. Standard curves were created and with Bio-Rad CFX Manager 3.1 software. Based on cq-values, the spore concentration in the samples was quantified, and calculated to genomes/g tissue.

#### Standard curve

To prepare the 5-point standard curves, 20 mg of tissue from uninfected mice were spiked either with 20 μL 0.9% NaCl as negative control or with 10^4^−10^8^ spores of *Lichtheimia corymbifera or Rhizopus arrhizus*. DNA was extracted from the 20 mg tissue or from 10^8^ spores as positive control using the DNeasy Blood & Tissue Kit. The final elution volume of 200 μL contained a DNA amount of 15–30 ng DNA. 2 μl of the DNA eluate were used in the PCR reaction as descripted above. The five point standard curve was established with three biological replicates and two technical replicates for each tissue type and fungus.

An R^2^ cut-off value for standard curves was applied at 0.9, thereby the values were little lower. The standards with best R^2^ values were taken as final standards for analyzing the samples (see S1 Fig). Fungal loads were determined with Bio-Rad CFX Manager 3.1 software using the 5-point standard curve as reference. Values are then converted to genome equivalents per gram of tissue for easy comparison.

More detailed data about the used PCR with the novel pan-Mucorales marker are given in [13].

### Statistical analysis

Statistical analyses were done using one-way ANOVA (Graph-prism software). Survival curves were compared using log-rank Mantel-Cox test (Graph-prism software). Values of *p* < 0.05 were considered as statistically significant.

## Results

### Dose-dependent survival of mice inhalatively infected with *Lichtheimia corymbifera* or *Rhizopus arrhizus*

*Lichtheimia corymbifera* and *Rhizopus arrhizus*, two prominent inducers of invasive mucormycosis, were used for all animal experiments. To evaluate the relevance of underlying risk factors and to compare the virulence of the two species, immunocompetent mice and animals with different underlying immunosuppressive regimens were inhalatively infected in order to mimic the most common entrance pathway. Two dosages of fungal spores were given, a lower dosage (ld) of 6x10^6^ CFU and a higher dosage (hd) of 1.2x10^7^ CFU.

**Fig 1:**
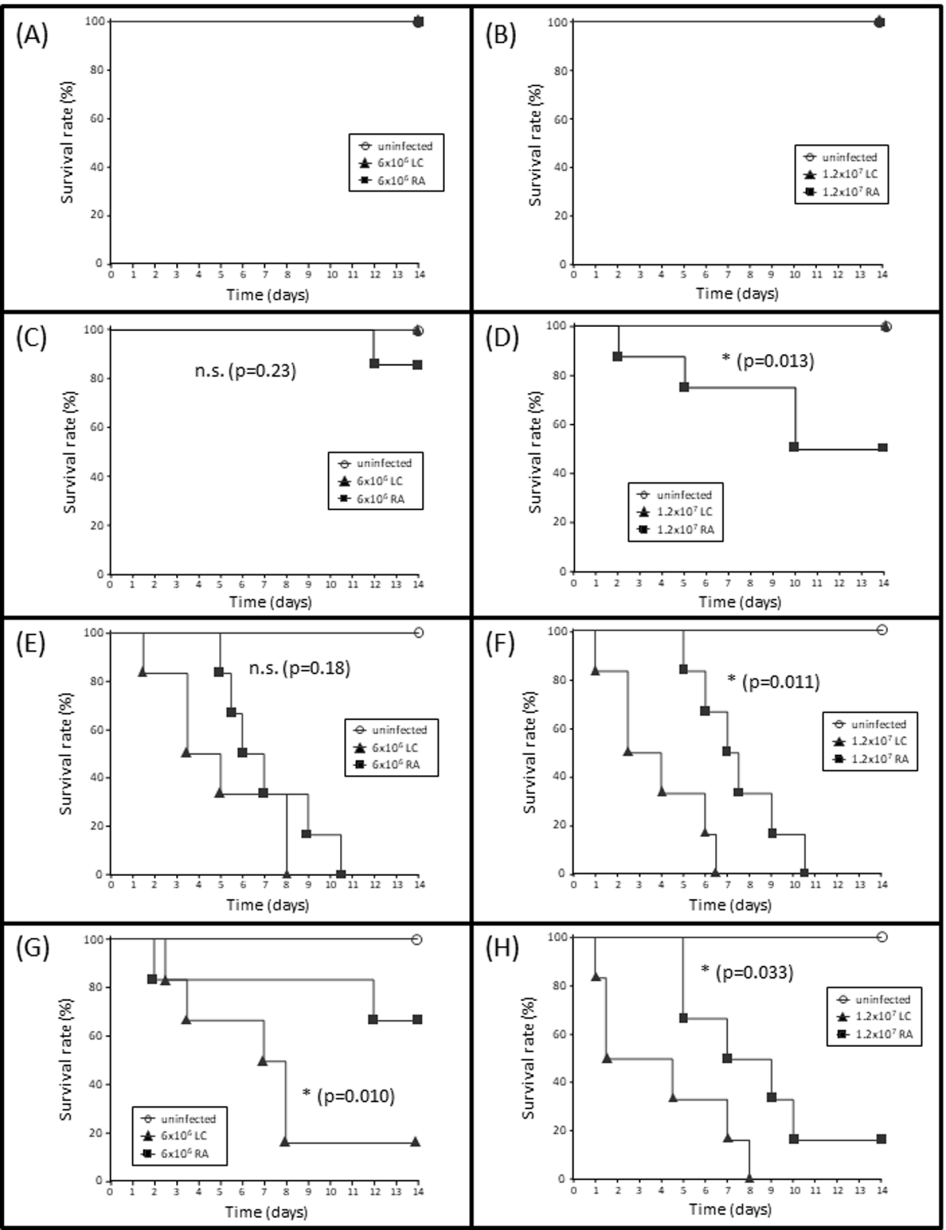
Survival curves after murine inhalative mucormycete infection. Wild type mice (A,B), diabetic mice (C,D) or mice treated with cortisone acetate (E,F) or cyclophosphamide (G,H) were inhalatively infected with a low dosage of 6x10^6^ (A,C,E,G) or a high dosage of 1.2x10^7^ (B,D,F,H) spores of *Lichtheimia corymbifera* (LC) or *Rhizopus arrhizus* (RA). Survival was monitored for 14d post infection. The survival curves for the different fungi were compared using log-rank Mantel-Cox test; * p<0.05; ** p<0.01; *** p<0.005.

Neither of the two fungal isolates did induce any mortality in the immunocompetent animals, not even if the higher spore dosage was used (Fig 1A and 1B). In diabetic mice, a certain dose-dependent mortality was visible for *Rhizopus arrhizus*, but not for the *Lichtheimia corymbifera* isolate. The difference in virulence between the two fungal species in diabetic animals reached statistical significance for the higher infectious dosage (Fig 1C and 1D).

Treatment with cortisone acetate (CA) or cyclophosphamide (CP) made the animals highly susceptible towards both mucormycete isolates and thus represented prominent risk factors (Fig 1E–1H). However, in contrast to the diabetic animals, *L*. *corymbifera* induced a higher mortality and faster disease progression than *R*. *arrhizus* for both pre-treatment conditions and for both inoculums. This tendency reached statistical significance in three out of four graphs (Fig 1F–1H).

### Clinical parameters of mice inhalatively infected with *Lichtheimia corymbifera* or *Rhizopus arrhizus*

**Fig 2:**
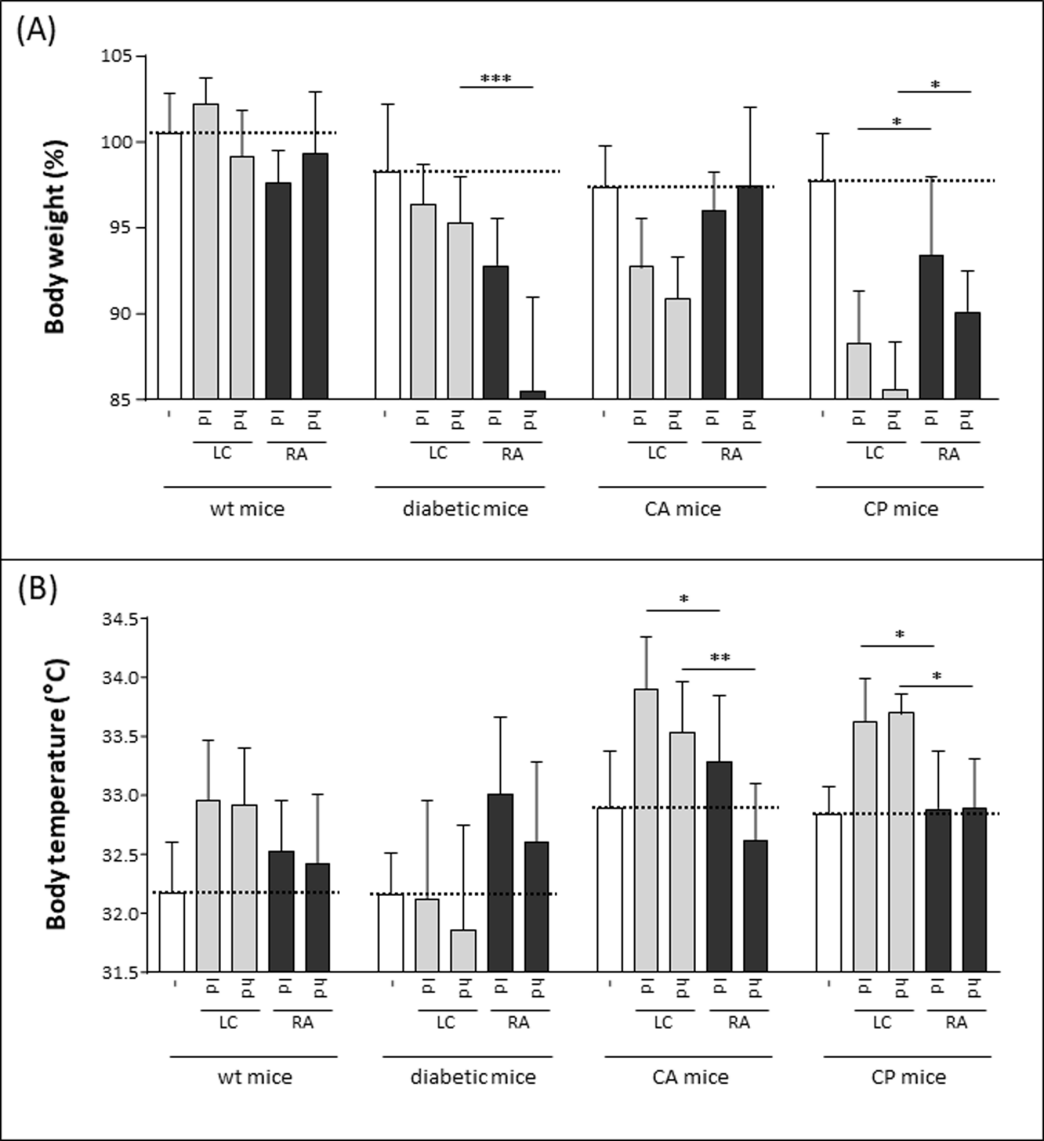
Body weight and temperature as clinical parameters for mucormycete infection. Wild type (wt) mice, diabetic mice or mice treated with cortisone acetate (CA) or cyclophosphamide (CP) were inhalatively infected with 6x10^6^ (ld) or 1.2x10^7^ (hd) spores of *Lichtheimia corymbifera* (LC) or *Rhizopus arrhizus* (RA). Body weight (A) and temperature (B) were controlled twice a day and are shown here for day 3 post infection. Body weight for the individual mice is calculated as % of body weight at day of infection. The different groups were statistically analyzed by one-way ANOVA; * p<0.05; ** p<0.01; *** p<0.005.

As induction of inappetence or anorexia in animals is an effect of inflammation and pro-inflammatory cytokines [14], we used the resulting weight loss as clinical indicator of the severity of mucormycete infection. This parameter of body weight loss confirms the difference in virulence between the two mucormycete strains and also the virulence dependency on the underlying immunosuppressive regimen (Fig 2A). The body weight of immunocompetent wild-type mice remained rather constant after infection, whereas diabetic mice slimmed considerably after infection with the *Rhizopus arrhizus* isolate, but not with *Lichtheimia corymbifera*. In contrast, the body weight of animals with cortisone acetate or cyclophosphamide treatment decreased more prominent after *Lichtheimia* infection than after *Rhizopus* infection; some of the differences between the fungal species even reached significance.

Since hyperthermia is another initial body response to infection, we used appearance of fever as further clinical parameter to quantify the severity of infection. A transient fever stage with body temperatures above the normal values was visible for all infected animals and reached its maximum for both fungal strains at day 3 (Fig 2B). Again, animals infected with *L*. *corymbifera* developed a more prominent fever compared to *R*. *arrhizus*-infected mice, indicating a higher virulence. Similar to the body weight, the only exception were diabetic animals, where *Rhizopus* induced higher fever than *Lichtheimia*.

The transient fever stage was followed by a stage of normal body temperature or of hypothermia when the animals lost the ability to maintain their temperature; the latter represented a humane endpoint (S2 Fig).

### Fungal load and dissemination after inhalative infection with *Lichtheimia corymbifera* or *Rhizopus arrhizus*

We aimed to analyze the fungal load in the lung as primary target and entrance site, both as a proof of successful infection and to study a putative correlation with virulence. For this purpose, we adapted an in-house established genetic marker based on the translation elongation factor (TEF) of mucormycetes for the quantification of genomic DNA in organ tissues gained from mice. Organ- and fungus-specific five-point standard curves were generated based on spiked-tissue samples (10^4^−10^8^ spores/20 mg tissue) to also include the impact of DNA extraction in the experimental setup. DNA extraction and quantitative PCR were very reproducible and accurate for spiked samples (S1 Fig), resulting in R² values of 0.842–0.993 depending on the organ and fungus. We were able to detect and quantify genomic DNA of mucormycetes in small quantities of murine organ homogenates (20 mg). The primers were found to be specific for mucormycetes [13].

**Fig 3:**
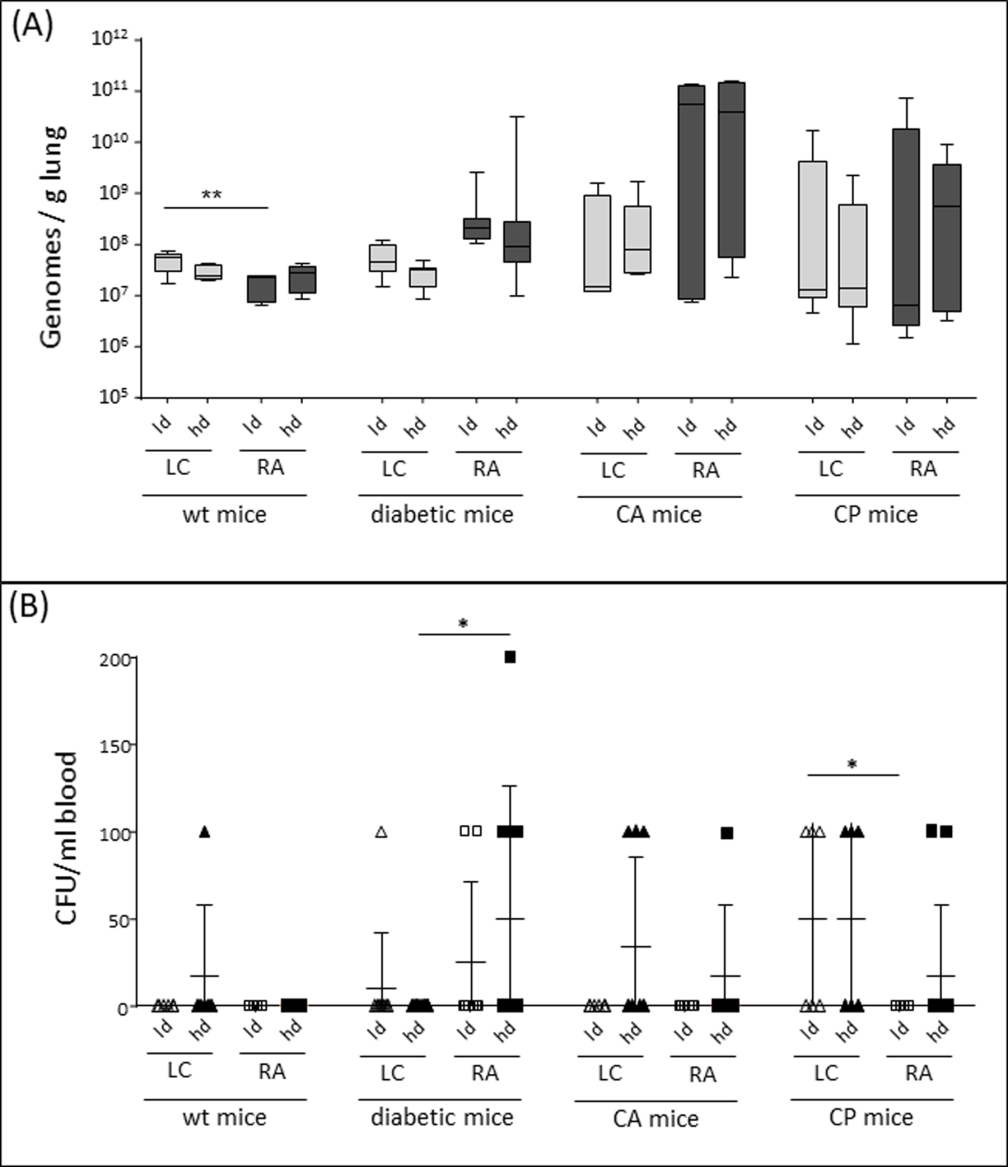
Fungal load in lung tissue and blood. Wild type (wt) mice, diabetic mice or mice treated with cortisone acetate (CA) or cyclophosphamide (CP) were inhalatively infected with 6x10^6^ (low dose; ld) or 1.2x10^7^ (high dose; hd) spores of *Lichtheimia corymbifera* (LC) or *Rhizopus arrhizus* (RA). (A) Fungal load in the lung was quantified by PCR at day of exitus. (B) Fungal dissemination was determined by CFU count in blood 24h post infection. The fungal load of the different groups was compared by one-way ANOVA; * p<0.05; ** p<0.01; *** p<0.005.

In our experiments, the two fungal isolates could be detected in all lung samples of the respective animal groups, proving the presence of the causative agents also in wild-type mice, which did not die in the course of the experiment (Fig 3A). Surprisingly, no statistically significant difference in fungal load was visible between the risk groups despite their variable susceptibility. In addition, only infected wild-type mice differed in fungal load between *Lichtheimia* and *Rhizopus* and thus reflected their different virulence.

Due to the limited correlation between survival curves and fungal load in the lung, we studied whether the differences in virulence of the fungi and susceptibility of the animals correlate with fungal dissemination. Quantification of colony-forming units in the blood taken 24h post infection revealed that the fungi had already started to disseminate into the circulation. In general, more fungal CFU were found in the blood of wt, CA-treated and CP-treated animals infected with the *Lichtheimia* isolate than in those infected with *Rhizopus* (Fig 3A). Only in the blood of diabetic animals, the fungal load was higher in *Rhizopus*-infected mice. This perfect correlation with the virulence pattern deduced from survival curves and clinical parameters indicates that the competence of dissemination and survival in the blood might be essential for the virulence of the mucormycetes.

**Fig 4:**
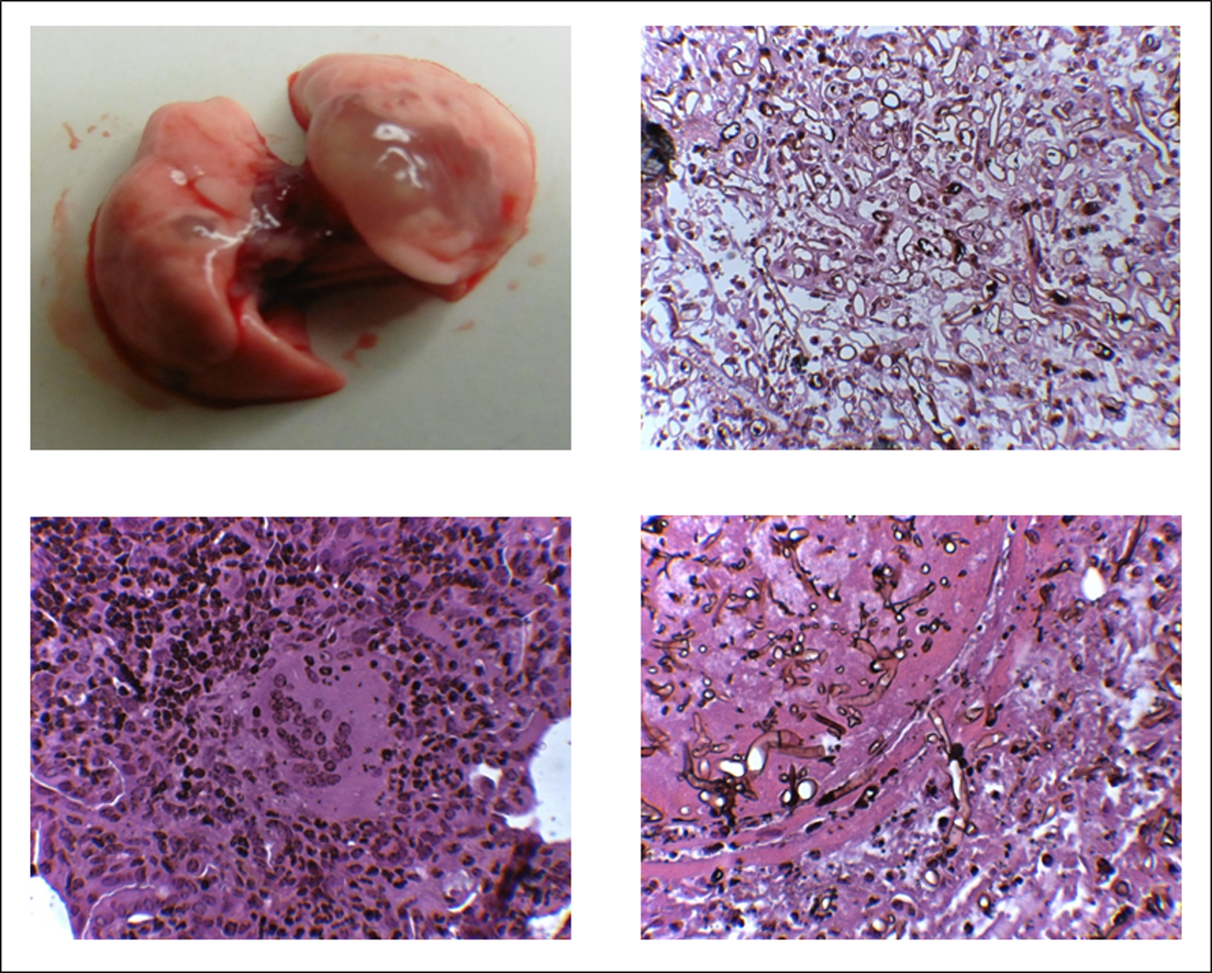
Typical macroscopic and microscopic changes of *Rhizopus arrhizus*-infected lungs. Wild-type mice, diabetic mice or mice treated with cortisone acetate or cyclophosphamide were inhalatively infected with 2x10^7^ spores of *Rhizopus arrhizus*. Lungs were dissected and sections were stained with a combination of hematoxylin-eosin and methenamine silver staining. Top, left: macroscopic view of a lung from a cyclophosphamide-treated animal infected with *R*. *arrhizus*; top, right: fungal hyphae in the lung tissue; bottom, left: multinucleated giant cells and infiltrating lymphocytes; bottom right: transmural penetration of fungal hyphae into a lung blood vessel.

Not only detection of fungal spores in the blood, but also the clinical symptoms of the animals suggested differences in dissemination between *L*. *corymbifera* and *R*. *arrhizus*. Mice infected with the *L*. *corymbifera* strain more often revealed symptoms of the central nervous system and/or inner ear involvement such as tremor, convulsions and vestibular dysfunctions than those infected with the *R*. *arrhizus isolate* (78% versus 41%, respectively). In contrast, macroscopic and microscopic changes of the lung were more prominent in *Rhizopus*-infected animals. An example is given in Fig 4. Macroscopic examination revealed extended glassy cystic-like parenchymal alterations (Fig 4 top left); microscopically, fungal hyphae, multinucleated giant cells and inflammatory cells were visible (Fig 4, top right and bottom left). In some single cases, transmural penetration from lung tissue into blood vessels could be revealed (Fig 4, bottom right).

### Response of *Lichtheimia corymbifera* and *Rhizopus arrhizus* to posaconazole prophylaxis

Since the capacity to induce breakthrough infections is a central parameter for virulence and high mortality in affected patients, the response of the two mucormycetes isolates towards posaconazole prophylaxis was examined. For that purpose, diabetic mice as well as mice treated with cortisone acetate or cyclophosphamide were studied after inhalative infection in the presence or absence of posaconazole. Immunocompetent wild-type mice were not included since our previous experiments did not show significant morbidity and mortality. Posaconazole prophylaxis started 5 days before infection, and daily application of the drug was continued throughout the complete experiment. To ensure local presence of posaconazole at the site of fungal entrance, drug concentrations were quantified in lung homogenates at the end of the experiment. Posaconazole could be detected in all lungs, and the local concentrations were between 2.6±1.8 ng/ml and 8.2±5.1 ng/ml; the lungs of CP exhibited even 19.1±9.1 ng/ml posaconazole.

**Fig 5:**
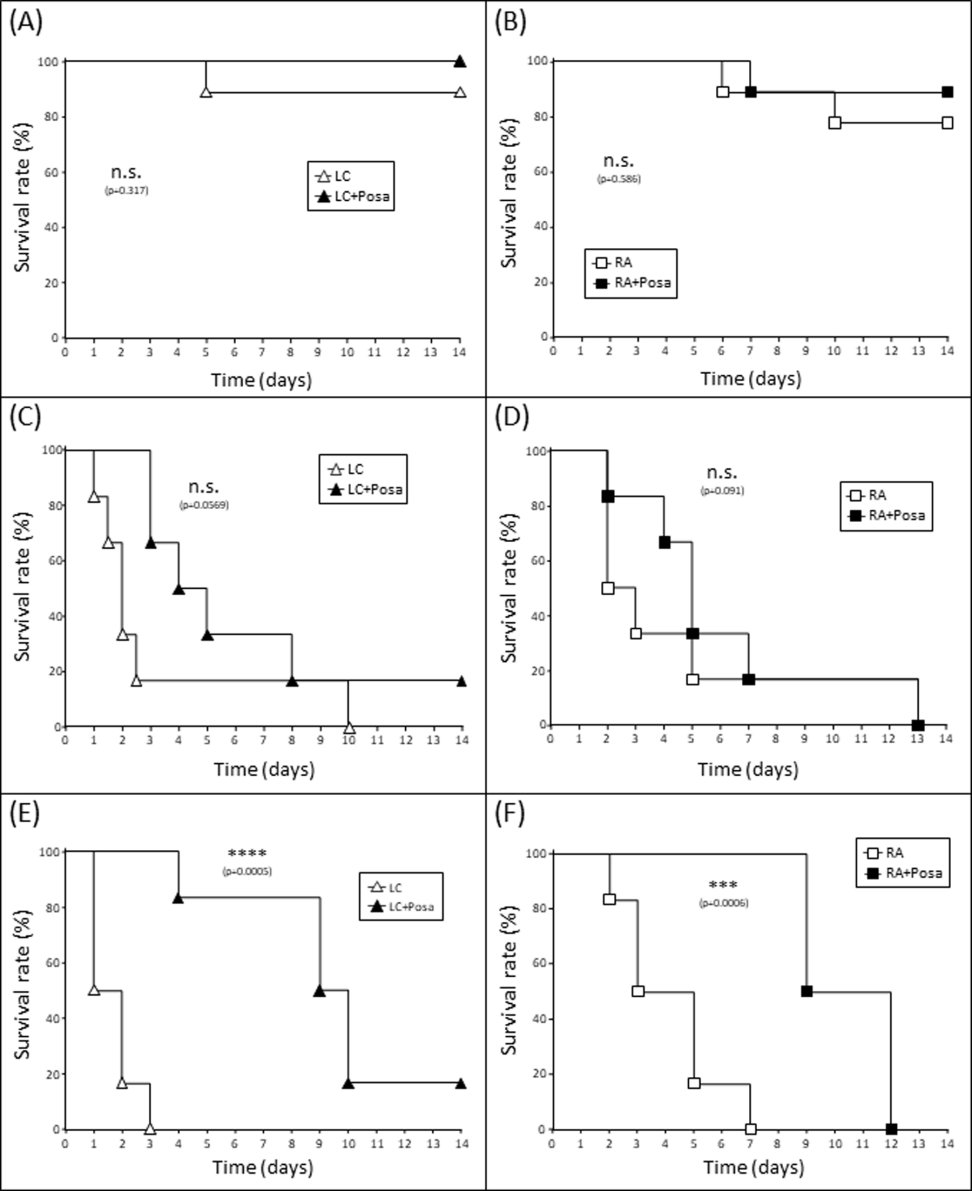
Survival curves of mice inhalatively infected with mucormycetes under posaconazole prophylaxis. Diabetic mice (A, B) or mice treated with cortisone acetate (C, D) or cyclophosphamide (E, F) were either mock-treated or treated with posaconazole. Inhalative infection with 2x10^7^ spores of *Lichtheimia corymbifera* (LC) (A, C, E) or *Rhizopus arrhizus* (RA) (B, D, F) was performed 5d after start of drug application. Survival was monitored for 14d post infection. The survival curves for the different fungi were compared using log-rank Mantel-Cox test; * p<0.05; ** p<0.01; *** p<0.005.

Both fungi were capable to induce breakthrough infections in animals receiving posaconazole prophylaxis (Fig 5A–5F). However, posaconazole delayed the onset of first symptoms and postponed mortality, at least in those animals that were immunosuppressed by cortisone acetate or cyclophosphamide. The protective effect of posaconazole was most prominent and highly significant in cyclophosphamide-treated mice (Fig 5E and 5F). Although the benefit of posaconazole prophylaxis was visible for both *Lichtheimia*- and *Rhizopus*-infected CP-treated mice, the profit was even higher for infection with the *Lichtheimia* isolate (Fig 5E).

### Detailed study of innate immunity in the pathogenesis of mucormycosis

We studied whether the number of circulating immune cells is modulated by mucormycetes infection depending on fungal species and on underlying immunosuppressive risk factor. In general, mice treated with CA and particularly those treated with CP showed a profound pancytopenia with low numbers of neutrophils, monocytes and lymphocytes, compared to wild type and diabetic mice, and regardless of infection. For the platelet number, no clear tendency was visible after mucormycete infection, but since the mean platelet volume (MPV) tends to be higher after infection, an increase in platelet renewal might take place (S3 Fig).

We aimed to investigate whether CA and CP treatment represent risk factors for lethal outcome of inhalative mucormycosis due to induced pancytopenia or due to selective neutropenia. For that purpose, we specifically induced neutropenia using a specific antibody, and compared the outcome of subsequent mucormycete infection with that of CA and CP-treated mice. For comparison, mice lacking other innate immune elements such as platelets or complement factor C3 were infected. Platelets harbor various immune functions, and thrombocytopenia represents a risk factor for invasive fungal infections [15, 16]; complement deficiency was tested since the complement system promotes T-cell priming and migration to the lung and is essential to control various infections [17, 18].

**Fig 6:**
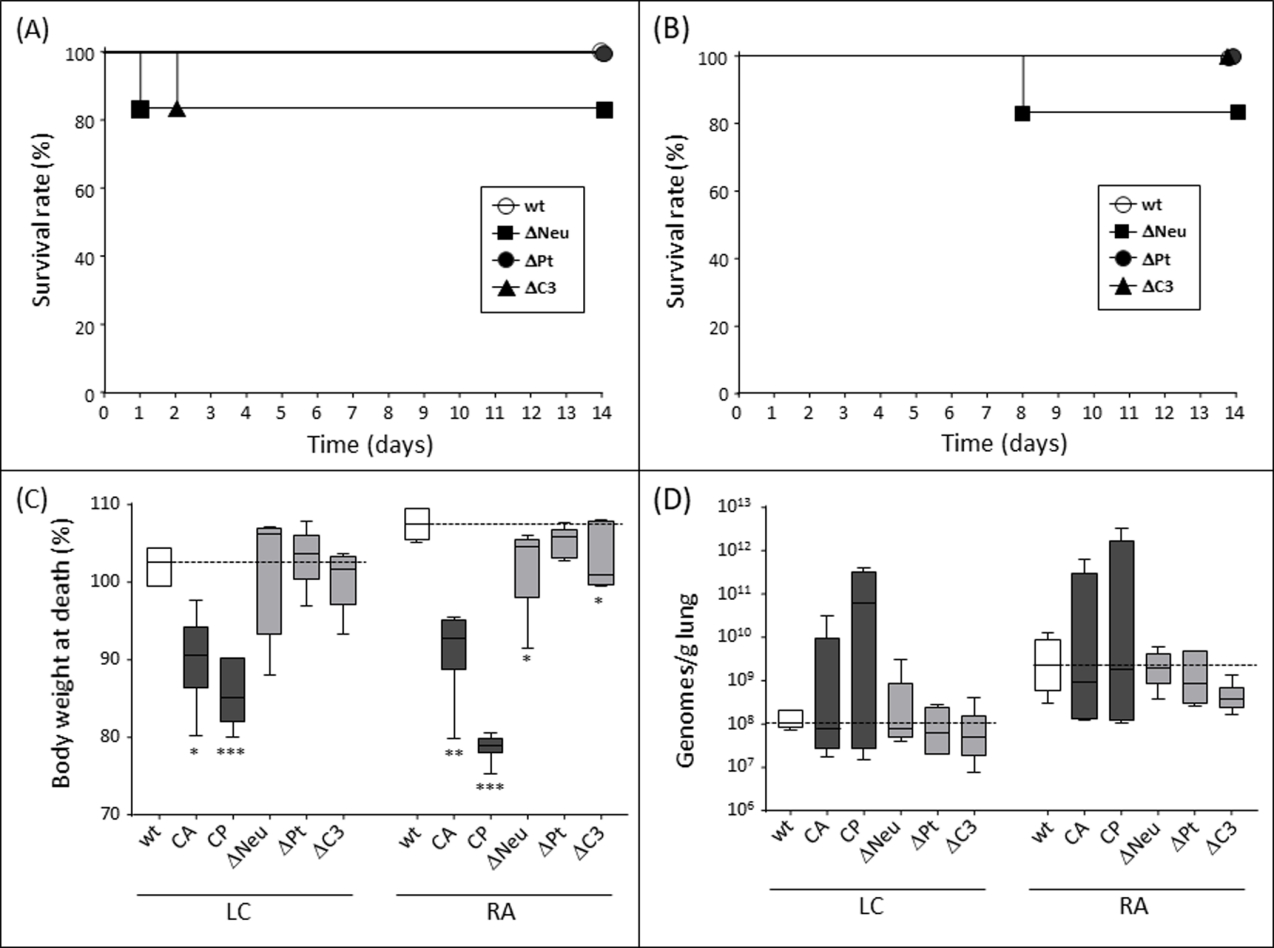
Survival, body weight and fungal load after mucormycete infection of mice with defined immunodeficiencies. Wild type (wt) mice or mice lacking neutrophils (ΔNeu), complement factor C3 (ΔC3) or platelets (ΔPt), were inhalatively infected with 2x10^7^ spores of *Lichtheimia corymbifera* (A, C, D) or *Rhizopus arrhizus* (B, C, D). (A, B) Survival was monitored for 14d post infection. (C) Body weight at time of death for the individual mice was calculated as percentage of body weight at day of infection. (D) Fungal load in the lung was quantified by PCR at day of exitus. * p<0.05; ** p<0.01; *** p<0.005.

Selective depletion of only neutrophils was not sufficient to make the animals highly susceptible against inhalative infection by the used isolates of *Lichtheimia corymbifera* or *Rhizopus arrhizus* (Fig 6A and 6B). Whereas all animals treated with CA or CP died in the course of the experiment (Fig 5), loss of 96% of the neutrophils, as achieved by the α-Ly6G antibody, was not reflected by a high mortality after infection with *Lichtheimia corymbifera* (Fig 6A) or *Rhizopus arrhizus* (Fig 6B). The survival curve of neutropenic animals was similar to that of mice deficient in complement factor C3, a central component of the complement system. These results indicate that either a broad pancytopenia or functionality loss of several immune cells is necessary for high susceptibility against mucormycetes. Alternatively, CP- or CA-induced depletion of immune cells other than neutrophils (such as alveolar macrophages) plays a predominant role. Interestingly, also selective thrombocytopenia did not modify the outcome of mucormycosis, although platelets are known to harbor a broad spectrum of innate immune functions [19] (Fig 6A and 6B).

The finding that selective neutropenia does not correlate with severity of mucormycosis was also reflected by body weight loss after infection. Whereas the body weight at time of death was significantly lower in CA- and CP-treated animals than in wild-type mice, the body weight of animals with selective neutropenia, thrombocytopenia, or deficiency of complement C3 was comparable to that of wild-type mice (Fig 6C). This tendency was visible for both *Lichtheimia corymbifera* and *Rhizopus arrhizus*. Similarly, the fungal load in the lungs of CA- or CP-treated mice was slightly higher than that in the lungs of wild-type mice or animals with neutropenia, thrombocytopenia or complement deficiency (Fig 6D). However, the differences in fungal load did not reach statistical significance.

## Discussion

Although mucormycosis is an emerging invasive infection with increasing incidence in immunocompromised individuals such as organ recipients or patients with hematological malignancies, knowledge about the differences in virulence, pathogenesis and dissemination among species is limited. Furthermore, no detailed analyses exist whether those parameters depend on the underlying risk factor of the patients. For that reason, we established a murine model for mucormycete infection with two prominent human pathogens, *Lichtheimia corymbifera* and *Rhizopus arrhizus*. The animals were infected inhalatively to mimic the most common entrance pathway of mucormycetes.

The murine model shows high incidence that treatment with corticosteroids or cytostatic drugs such as cyclophosphamide are central risk factors to acquire mucormycosis. Interestingly, infection with *Rhizopus arrhizus* correlated with delayed clinical course and, at least partly, better outcome compared to the *Lichtheimia corymbifera* strain. This is in striking contrast to *in vitro* experiments, where *Rhizopus arrhizus* showed faster growth and sporulation (S4 Fig). One putative reason for the lower virulence of *R*. *arrhizus* compared to *L*. *corymbifera* might be the spore size: the spore diameter of *Lichtheimia corymbifera* is rather small with 2.8–4.2 μm, compared to the larger spores of *Rhizopus arrhizus* with 5.5–7.8 μm [10]. It might be hypothesized that the smaller *Lichtheimia* spores are deeply inhaled by the animals and reach the lung alveoli, where they start to germinate and disseminate into the blood stream. In contrast, the larger *Rhizopus* spores preferentially remain in the upper airways and only reach the alveoli and the bloodstream after prolonged incubation time. This hypothesis might be supported by the fact that neurological symptoms, which are indicators for fungal penetration into the central nervous system, were more often observed in mice infected with *Lichtheimia* than in those infected with the *Rhizopus* isolate (S5 Fig).

However, our experiments indicate that susceptibility to mucormycosis strictly depends on underlying immunosuppression. In contrast to treatment with cortisone acetate or cyclophosphamide, induction of diabetes made the animals highly susceptible to infection by the R. arrhizus isolate, but represented only a minor risk factor for infection with the L. corymbifera strain. One reason for that could be that the pathogenesis of *Rhizopus* infection, as discussed above, is mainly concentrated on the respiratory tract, whereas *Lichtheimia* with its smaller spores quickly disseminates. Consequently, the impairment of alveolar macrophages by diabetes and high glucose levels [20, 21] would mainly affect the *Rhizopus*-induced mucormycosis and result in higher fungal load in the bloodstream (see Fig 3B). Our results might reflect the epidemiology of mucormycosis in India, where uncontrolled diabetes represents a major risk factor, and where *Rhizopus arrhizus* (formerly called *Rhizopus oryzae*) is the most common inducer of mucormycosis [22].

Posaconazole is one of only three antimycotic drugs that are currently approved for the salvage therapy of mucormycosis [23]. Only limited data exist about the species specificity of responsiveness to posaconazole treatment. Our experiments show that posaconazole prophylaxis does not completely protect from disease onset, although posaconazole levels were clearly detectable in the lung of the animals. However, the course of disease is significantly delayed for both included isolates of *Lichtheimia* and *Rhizopus*. Interestingly, we found prominent differences between the underlying risk factors: the protecting and disease-delaying effect of posaconazole is minor or not present in diabetic mice, only minor in animals treated with cortisone acetate, and highly significant in cyclophosphamide-treated mice. Further experiments will aim to evaluate the underlying mechanisms.

Platelets harbor a variety of innate immune functions, and their precise position in the immune network attracted mounting attention in the last years [19]. For a number of fungal species as well as for bacterial and viral infections, platelets are well established players of the immune defense [24]. They directly attack pathogens, but also orchestrate the antimicrobial immune network. Several reports mention low platelet counts and thrombocytopenia to promote invasive fungal infections [15, 16]. In addition, platelet parameters could be utilized as prognostic markers in the setting of neonatal fungal infections [25] and are predictive for the outcome of aspergillosis in neutropenic patients [26]. In our study, we found that platelet deficiency does not increase the susceptibility to mucormycosis. These results might imply that they play a minor role in the defense against mucormycetes. Alternatively, the loss of immune functionality in thrombocytopenia might be counterbalanced by reduced risk of thrombosis. Since thrombosis is a common finding in mucormycosis, the latter explanation cannot be excluded and awaits further research.

Neutropenia is commonly considered a main risk factor for invasive fungal infections. However, treatment with cortisone acetate or cyclophosphamide does not only induce neutropenia, but also affect generation and functionality of other immune cells. A precise analysis in our murine model indicates that isolated neutropenia is not sufficient to make the animals highly vulnerable for infections caused by *Lichtheimia* or *Rhizopus*. Since the number of neutrophils in the uninfected lung is low, it seems reasonable to argue that neutrophils are not crucial for the first-line defense against airborne pathogens, but are the major immune cells in the advanced stage of lung infection and/or after penetration of the pathogens into the bloodstream. As implied by the diabetes results, number and functionality of alveolar macrophages might be the central parameters in the very early stages of respiratory tract infections caused by mucormycetes. Future experiments will aim to deplete this cell type and thus highlight their role in the course of disease.

Taken together, our experiments yielded interesting findings showing considerable differences between two isolates of *R*. *arrhizus* and *L*. *corymbifera*, which are important causes of mucormycosis. These differences include fungal virulence, susceptibility of the host by specific risk factors, symptoms and progression of the disease and response to antimycotic therapy. However, there may also be substantial intraspecies variabilities that we could not address by now. Since our experiments include a large variety of different experimental conditions and animal groups, insights about the intraspecies heterogeneity have to remain limited also in the future due to ethical reasons. Other reports support the hypothesis of heterogeneity in virulence and phagocytic vulnerability [27, 28] and imply that the geographical origin of the isolates might play a substantial role. In summary, mucormycosis appears to be a very heterogeneous disease, and a lot of scientific research remains to be done for a deeper insight of interactions of the various fungal agents and isolates with the host defense, which will be a vital prerequisite for new therapeutic options.

## Supporting information

S1 Fig Five-point-standard curves for organ- and fungus-specific quantification of fungal load. TEF was used as genetic marker. (A) represents the standards of kidney inoculated with *Lichtheimia corymbifera*. (B) is the curve of brain and *Lichtheimia corymbifera* and (C, D) show the lung tissue standards with inoculated *Lichtheimia corymbifera* (C) and *Rhizopus arrhizus* (D).
(TIF)

S2 Fig Body temperature of animals at the end of experiment (survivors) or at reaching a humane endpoint (exitus). Temperature was measured ventrally using a non-contact thermometer.
(TIF)

S3 Fig Blood count at day 2 after start of experiment. Wild type (wt) mice or animals treated with cortisone acetate (CA) or cyclophosphamide (CP) were mock-infected or inhalatively infected with 2x10^7^ spores of *Lichtheimia corymbifera* (LC) or *Rhizopus arrhizus* (RA). Blood count was evaluated at day 2 after start of experiment. The parameters of infected mice were statistically compared with the numbers of uninfected animals by one-way ANOVA; * p<0.05; ** p<0.01; *** p<0.005.
(TIF)

S4 Fig In vitro growth rate of Lichtheimia Corymbifera (LC) and Rhizopus Arrhizus (RA). Spores of both isolates were inoculated on SUP plates and incubated for up to 48h at either 30°C or 37°C. Photos were taken at indicated time points.
(TIF)

S5 Fig Frequency of animals with neurological symptoms after infection with Lichtheimia Corymbifera (LC) or Rhizopus arrhizus (RA). 107 LC-infected and 101 RA-infected animals were investigated for appearance of circling and movement disorders.
(TIF)
