# Supplementary figures and images for: Comparative immunopathogenesis in a murine model of inhalative infection with the mucormycetes Lichtheimia corymbifera and Rhizopus arrhizus
Source: PLoS One. 2020 Jun 17;15(6):e0234063. doi: 10.1371/journal.pone.0234063 (PMC7299637; doi:10.1371/journal.pone.0234063)

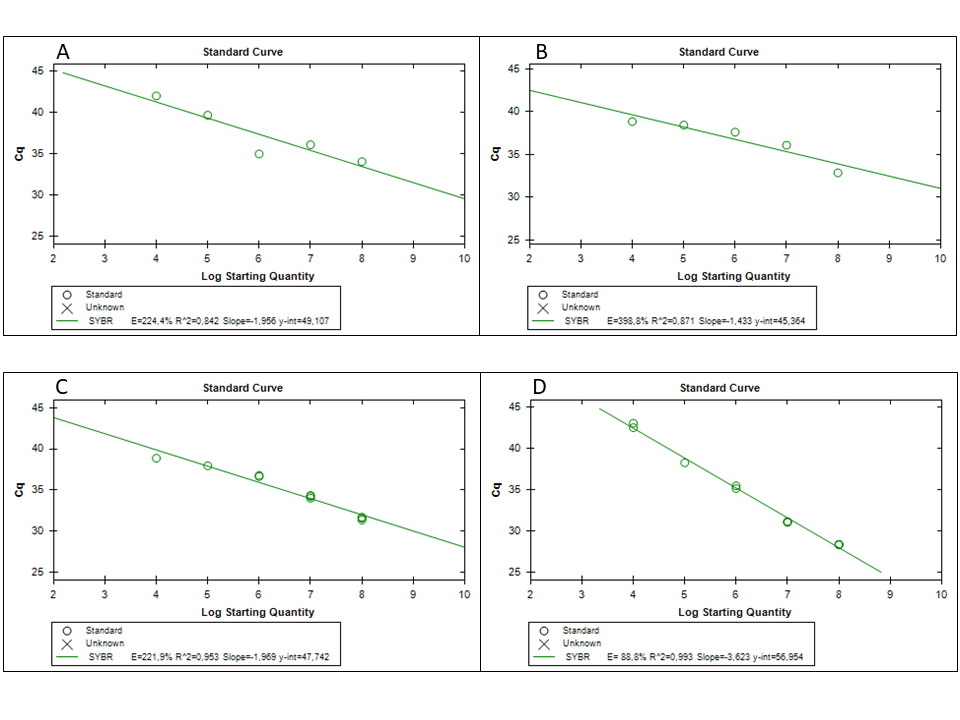

Supplement: S1 Fig — TEF was used as genetic marker. (A) represents the standards of kidney inoculated with Lichtheimia corymbifera. (B) is the curve of brain and Lichtheimia corymbifera and (C, D) show the lung tissue standards with inoculated Lichtheimia corymbifera (C) and Rhizopus arrhizus (D). (TIF) [file pone.0234063.s001.tif]

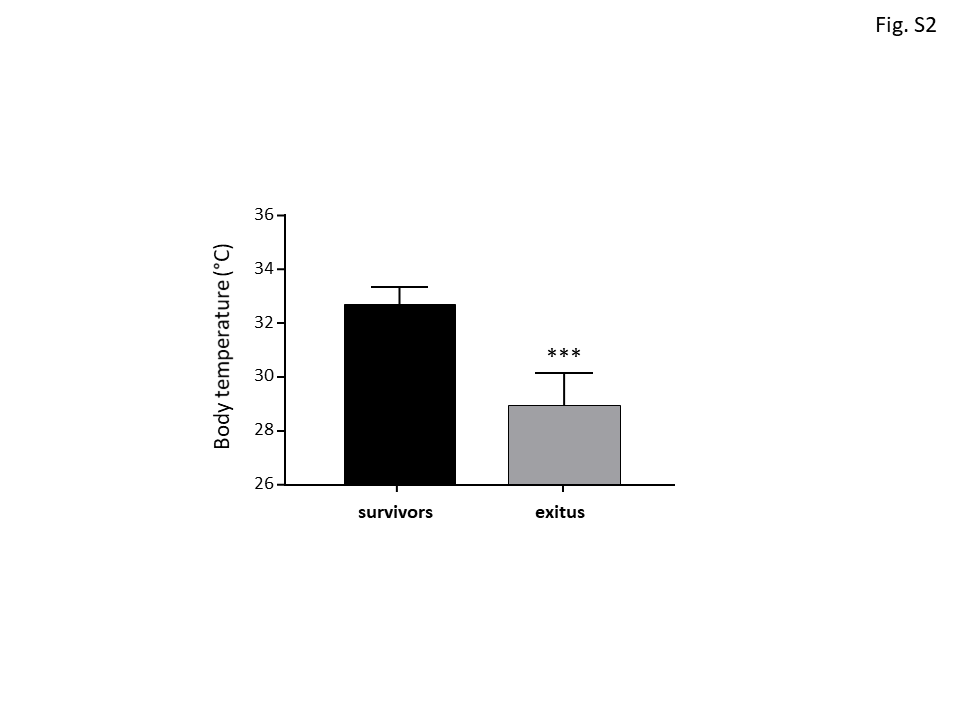

Supplement: S2 Fig — Temperature was measured ventrally using a non-contact thermometer. (TIF) [file pone.0234063.s002.tif]

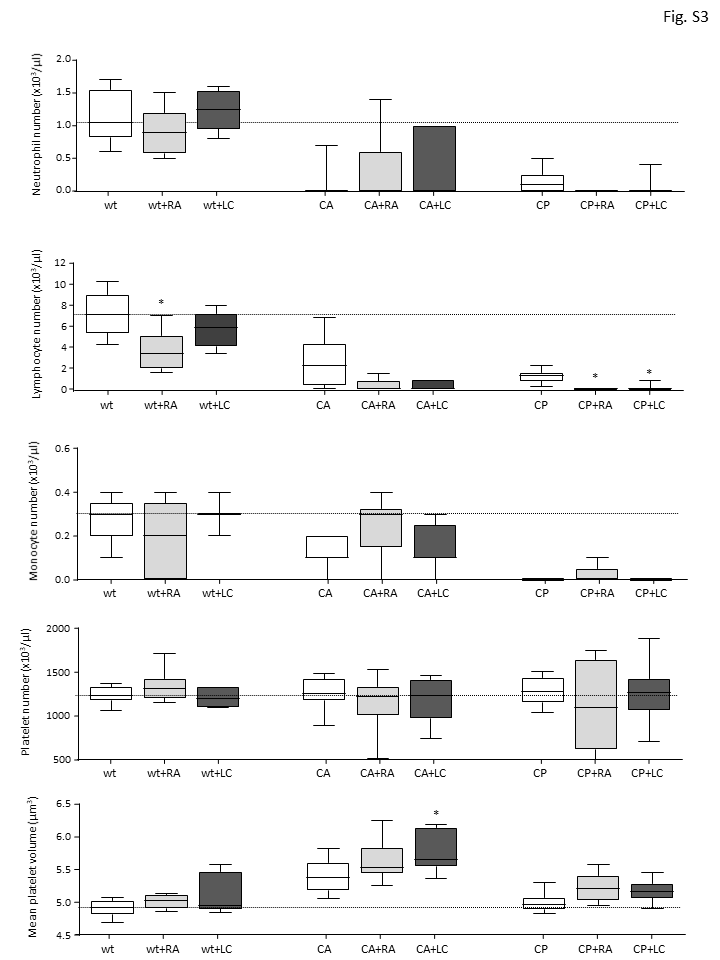

Supplement: S3 Fig — Wild type (wt) mice or animals treated with cortisone acetate (CA) or cyclophosphamide (CP) were mock-infected or inhalatively infected with 2x10^7^ spores of Lichtheimia corymbifera (LC) or Rhizopus arrhizus (RA). Blood count was evaluated at day 2 after start of experiment. The parameters of infected mice were statistically compared with the numbers of uninfected animals by one-way ANOVA; * p<0.05; ** p<0.01; *** p<0.005. (TIF) [file pone.0234063.s003.tif]

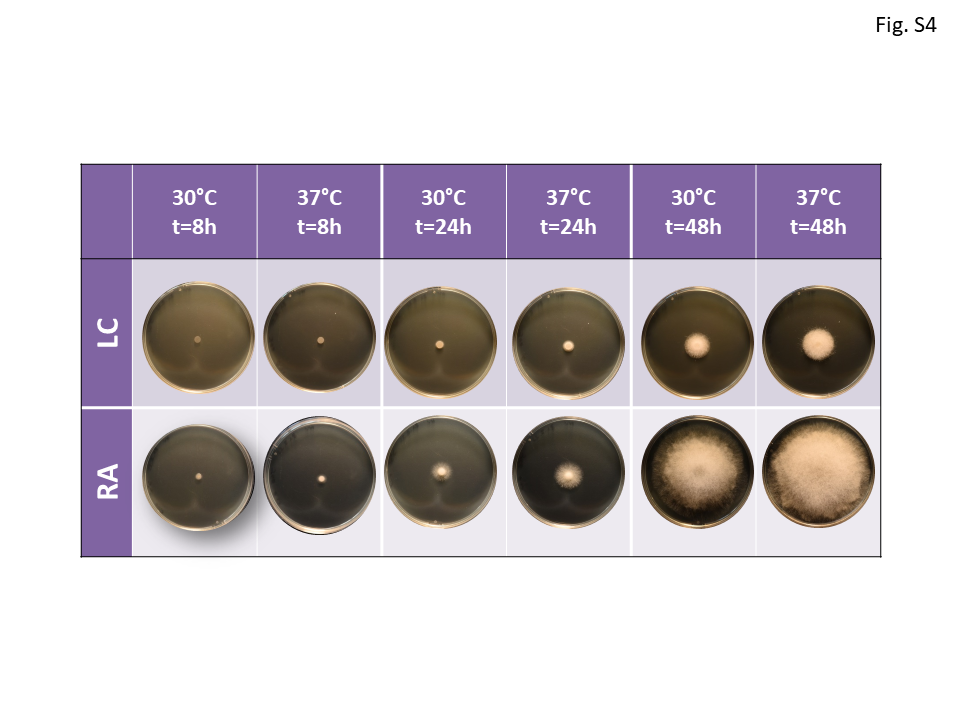

Supplement: S4 Fig — Spores of both isolates were inoculated on SUP plates and incubated for up to 48h at either 30°C or 37°C. Photos were taken at indicated time points. (TIF) [file pone.0234063.s004.tif]

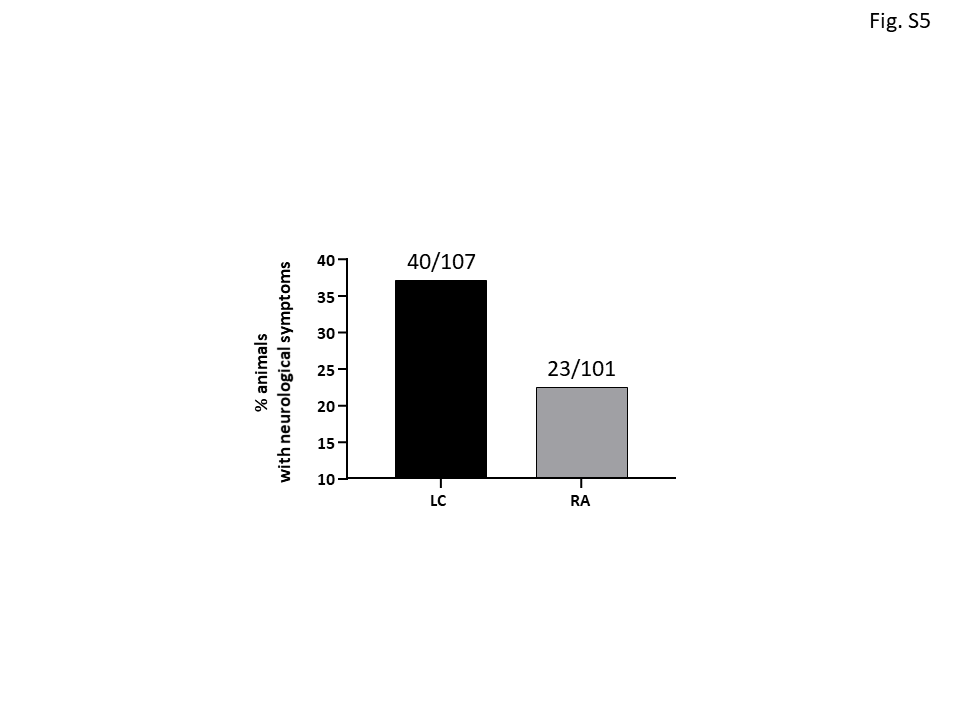

Supplement: S5 Fig — 107 LC-infected and 101 RA-infected animals were investigated for appearance of circling and movement disorders. (TIF) [file pone.0234063.s005.tif]
